# Multiple perinatal characteristics affect the association between maternal diabetes status and early neonatal gut microbiota

**DOI:** 10.1128/msphere.00914-24

**Published:** 2025-05-16

**Authors:** Cheng Liu, Wei Zheng, Jia Wang, Xianxian Yuan, Yuan Zhang, Yuanyuan Wang, Xu Ma, Guanghui Li

**Affiliations:** 1 Department of Obstetrics, Beijing Obstetrics and Gynecology Hospital, Capital Medical University, Beijing Maternal and Child Health Care Hospital, Beijing, China; 2 National Research Institute for Family Planning, Beijing, China; 3 National Human Genetic Resources Center, Beijing, China; University of Michigan-Ann Arbor, Ann Arbor, Michigan, USA

**Keywords:** gut microbiota, gestational diabetes mellitus, perinatal characteristics, meconium, cohort study

## Abstract

Increasing evidence has suggested that maternal gestational diabetes mellitus (GDM) can influence the neonatal gut microbiota. However, the initial microbial colonization of neonates is still unclear. The discrepancy in results between studies may be due to many other prenatal characteristics. This study aimed to investigate whether perinatal characteristics affect the association between maternal GDM status and early neonatal gut microbiota. This nested case-control study was based on a cohort of mothers and children (2016YFC1000304). Meconium samples were collected from neonates of mothers with (*n* = 114) and without GDM (*n* = 133) within 24 h after birth, and then assessed via 16S rRNA gene amplicon sequencing. Differences in the diversity and composition of the neonatal gut microbiota were compared according to maternal GDM status and other perinatal characteristics. The gut microbiota of neonates born to mothers with GDM presented lower alpha diversity with the Chao1 index (*P* = 0.0235). Principal coordinate analysis revealed that the meconium samples were clustered by maternal GDM status only with unweighted UniFrac distances (*R*^2^ = 0.011, *P* = 0.003). In other groups, such as maternal age ≥ 35 years old and maternal prepregnancy BMI ≥ 24 kg/m^2^, meconium was not clustered by maternal GDM status. Linear discriminant analysis revealed that 81 taxa were significantly different between the GDM group and the control group. Based on delivery mode, there were 226 representative taxa in the control group, whereas in the GDM group, there were no representative taxa. In addition, based on neonatal sex, there were 79 representative taxa in the GDM group and seven in the control group. Other perinatal characteristics, such as maternal prepregnancy BMI, age, gestational weight gain, and birth weight also influenced the differential taxa of the neonatal gut microbiota between the two groups. In our cohort, newborns from mothers with GDM and without GDM had similar composition but different abundances of the gut microbiota. Maternal prepregnancy BMI, age, gestational weight gain, and neonatal delivery mode, sex, and birth weight had different influences on the diversity and differential taxa of the neonatal gut microbiota. The results of this study suggest that when studying the association between GDM and neonatal gut microbiota, it is necessary to consider the concomitant perinatal characteristics.

**IMPORTANCE:**

This study uses 16S rRNA gene amplicon sequencing to analyze 247 meconium samples with or without maternal gestational diabetes mellitus (GDM) and make a multi-group comparison. We found that newborns from mothers with GDM and normoglycemic mothers had similar compositions but different abundances of the gut microbiota. Other than the maternal diabetes status, maternal body mass index, age, gestational weight gain, and neonatal delivery mode, gender and birth weight all contribute to neonatal gut microbiota. Compared with former related studies, our sample size was larger, and meconium was collected within 24 h after birth to avoid the influence of the living environment, feeding methods, mother's lifestyle, or diet. The results of this study will provide some information on the association between maternal GDM and neonatal gut microbiota colonization in early life and highlight the influence of non-negligible concomitant perinatal characteristics.

## INTRODUCTION

Gestational diabetes mellitus (GDM) is a glucose intolerance disorder that develops or is first recognized during the second or third trimester (1). Epidemiological and experimental data reveal a strong link between maternal metabolic health and the origins of obesity and metabolic disorders in subsequent generations (2). In particular, GDM increases the risk of obesity, type 2 diabetes, and metabolic syndrome in offspring (3–5). The mechanism by which maternal diabetes increases the risk of these chronic diseases in offspring is not well understood. A new hypothesis is that this effect may be mediated by changes in the maternal microbiome during pregnancy, and these changes may affect the newborn during pregnancy, delivery, or after birth.

The primary colonization of the human microbiota is currently poorly understood, and cumulative evidence suggests the occurrence of microbial vertical transmission from mothers to babies (6–9). The gut microbiota of pregnant women is affected by several factors and changes dynamically during pregnancy. Therefore, many scientists believe that maternal health and prenatal characteristics can influence intergenerational microbiota transmission and contribute to initial microbial colonization (6, 10, 11). The composition of the human gut microbiota in early life may affect risk factors related to health conditions in adulthood (12, 13).

Studies on the microbiota in neonates have shown differences in microbial composition between infants born to mothers with and without GDM (14–16). Furthermore, many studies have reported that specific taxa associated with GDM can be transmitted to offspring and differentiate their gut microbiota from those of offspring born to normoglycemic women (14, 17, 18). However, some studies reported that there was no difference in the neonatal gut microbiota between mothers with GDM and their non-GDM counterparts (19). The results of studies on the effects of maternal GDM on the neonatal gut microbiota have varied widely. These studies are often confounded by various perinatal conditions known to disrupt offspring microbiota colonization, such as mode of delivery, antibiotic usage, maternal body mass index (BMI), or gestational weight gain. Furthermore, neonates are quickly colonized by all kinds of microorganisms from outside after birth. Variability in the control of confounders in these studies raises the need for caution when interpreting and comparing published data while illustrating the need for further investigations to determine how to best characterize the gut microbiota in infants born to women with GDM.

In this study, we focused on the microbiota of meconium, which directly reflects the primary condition after birth, to provide a comprehensive understanding of potential factors involved in initial microbial colonization. We aimed to evaluate the influence of maternal diabetes status on the neonatal gut microbiota and investigate whether other perinatal characteristics affect the influence of GDM.

## MATERIALS AND METHODS

### Participant recruitment

This nest case-control study was based on a mother and child cohort (2016YFC1000304) in Beijing Obstetrics and Gynecology Hospital, Capital Medical University. The study was approved by the Ethics Committee of Beijing Obstetrics and Gynecology Hospital (2017-KY-015-01). Written informed consent was obtained from every participant. Written informed consent was also obtained from the parent or legal guardian of all minors participating in the study. All procedures were performed in compliance with the Declaration of Helsinki. The meconium samples in this study were collected from August 2018 to December 2019. We selected the participants according to the characteristics of the mothers. The inclusion criteria were as follows: (i) pregnant women whose weeks of gestation were 37 to 42 weeks; (ii) pregnant women with GDM or without GDM; (iii) pregnant women with singleton pregnancy; and (iv) pregnant women with complete clinical information. The exclusion criteria were as follows: (i) pregnant women with any other complications, such as hypertension disorders, disease of the digestive tract, or infectious diseases; (ii) pregnant women using any type of antibiotic during pregnancy, including the antibiotic administered before cesarean section; (iii) newborns administered any type of antibiotic after birth before collecting the samples; and (iv) newborns with major congenital anomalies. GDM was diagnosed at 24–28 gestational weeks according to the American Diabetes Association criteria (20), and a 75 g oral glucose tolerance test (OGTT) was performed. Pregnant women were diagnosed with GDM if one or more of the following applied glucose levels were elevated: fasting ≥ 5.1 mmol/L, 1 h ≥ 10.0 mmol/L, 2 h ≥ 8.5 mmol/L. Pregnant women diagnosed with diabetes or impaired glucose tolerance in the first trimester were also excluded. Pre-pregnancy BMI classification and gestational weight gain (GWG) were determined according to the Guidelines for Prevention and Control of Overweight and Obesity in Chinese. Underweight was defined as BMI < 18.5 kg/m^2^; normal weight was defined as BMI ≥ 18.5 and <24 kg/m^2^; overweight was defined as BMI ≥ 24 and <28 kg/m^2^ ; and obesity was defined as BMI ≥ 28 kg/m^2^. Controls were recruited from the same cohort and matched to the GDM cases concerning maternal age (±3 years) and pre-pregnancy BMI.

### Sample collection

Approximately 100 mg of meconium was taken from the diaper and transferred to a PSP Spin Stool DNA Plus Kit (Stratec Biomedical, Birkenfeld, Germany) following a standardized procedure. The samples were then shipped immediately to the laboratory on dry ice and stored in a freezer at −80°C. All the samples were collected within 24 h after birth in duplicate.

### Meconium DNA extraction and 16S rRNA gene sequencing

Genomic DNA was extracted using an MN NucleoSpin 96 Soi (MACHEREY-NAGEL, Germany) according to the manufacturer’s protocols. DNA isolation controls were also established and sequenced. The hypervariable V3–V4 region of the bacterial 16S rRNA genes was amplified using primers 341F (5′-CCTACGGGRSGCAGCAG-3′) and 806R (5′- GGACTACVVGGGTATCTAATC-3′). The PCR was performed in a 10 μL mixture containing 5 μL of KOD FX Neo Buffer, 0.3 μL of each primer (10 µM), 2 μL of dNTP (2 mM each), and 50 ng of meconium genomic DNA. The PCR amplification program included initial denaturation at 95°C for 5 min, followed by 25 cycles at 95°C for 30 s, 50°C for 30 s, and 72°C for 40 s, and a final extension at 72°C for 7 min. We conducted two PCRs for each sample and combined them after PCR amplification. Simultaneously, we established a PCR negative control. Amplicons were gel-purified and quantified using a NanoDrop spectrophotometer (NanoDrop Technologies) and Qubit 2.0 (Invitrogen, MA, USA). Finally, all PCR products were quantified by using Quant-iT dsDNA HS reagent and pooled together. TruePrep Index Kit V2/V3/V4 for Illumina (Vazyme, TD202-207) was used to prepare the library, then sequencing was performed on a HiSeq 2500 platform to generate paired-end reads of 250 bp (Illumina, CA, USA). DNA extraction, library construction, and sequencing were conducted at the Annoroad Genome Technology company (Beijing, China).

### Descriptive analysis of general characteristics

Statistical analyses were performed via GraphPad Prism 8.0. The general characteristics of the participants in the two groups were compared via a two-tailed *t*-test for normally distributed continuous variables, the Wilcoxon rank-sum test for non-normally distributed continuous variables, and the *χ* test or Fisher’s exact test for categorical variables. *P* < 0.05 was considered statistically significant.

### Bioinformatics analyses

The paired-end sequences were merged as raw tags via FLASH (version 1.2.11) (21). These raw tags were then strictly quality filtered via Trimmomatic (version 0.33) (22), and chimera sequences were removed via UCHIME (version 4.2) (23). High-quality reads were clustered into operational taxonomic units (OTUs) via USEARCH (version 10.0) (24) with a similarity threshold of 97%. Taxonomy was assigned to individual OTUs via the RDP classifier based on Silva (release 128, http://www.arb-silva.de) and Unite (release 7.2, http://unite.ut.ee/index.php) databases. All the samples were randomly subsampled to equal depths of 25,750 reads before the calculation of alpha and beta diversity metrics. Low-abundance taxa were removed if below the relative abundance cutoff of 0.005%.

Alpha diversity was assessed via the Shannon and Chao1 richness indices. Beta diversity was estimated via principal coordinate analysis (PCoA) analyses and generated with the binary Jaccard, Bray Curtis, weighted UniFrac, and unweighted UniFrac distances. The dissimilarity of the groups was assessed via permutational multivariate analysis of variance (PERMANOVA) and ANOSIM. *R*^2^ and *P* are from the PERMANOVA as implemented in the adonis function of the vegan R package.

The linear discriminant analysis (LDA) score, LDA effect size (LEfSe) analysis (LDA score > 2), the Wilcoxon signed-rank test, and balance tree analysis were employed to analyze microbial transmission. The false discovery rate was calculated using the Benjamini and Hochberg method (p.adjust function in R). For the Wilcoxon signed-rank test, the abundance data were subjected to a centered log-ratio transformation.

## RESULTS

### Characteristics of the participants

A total of 247 participants who had complete sequencing results were included in this study. Following the study design, 114 newborns from mothers with GDM and 133 from mothers without GDM were analyzed to explore the differences in the neonatal gut microbiota. As shown in Table 1, mothers with GDM had greater gravidity and parity. Although the prepregnancy weight and BMI did not differ between the two groups, the control group had much greater GWG. We also compared maternal lipid parameters in the third trimester, and only the high-density lipoprotein (HDL) level was greater in the control group. Other maternal characteristics did not differ between the groups. There were no significant differences in the mode of delivery, birth weight, or neonatal sex between the GDM and control group.

**TABLE 1:** Clinical characteristics of the participants^*a*^^,*b*^

Characteristic	GDM (*n* = 114)	Control (*n* = 133)	*P*
Maternal			
Age, years, mean ± SD	33.84 ± 3.75	33.56 ± 3.68	0.5576
Gravidity, mean ± SD	2.26 ± 1.10	1.88 ± 1.00	0.0050**
Parity, mean ± SD	1.51 ± 0.52	1.33 ± 0.52	0.0064**
Pre-weight, kg, mean ± SD	58.50 ± 6.36	61.81 ± 10.94	0.7528
PPBMI, kg/m^2^, mean ± SD	23.44 ± 3.76	23.28 ± 3.75	0.7460
Underweight, *n* (%)	7 (6.1%)	10 (7.5%)	0.6697
Normal weight, *n* (%)	66 (57.9%)	71 (53.4%)	0.4770
Overweight, *n* (%)	27 (23.7%)	35 (26.3%)	0.6344
Obese, *n* (%)	14 (12.3%)	17 (12.8%)	0.9056
GWG, kg, mean ± SD	11.60 ± 4.90	13.97 ± 4.40	<0.0001****
OGTT, mmol/L, mean ± SD			
Fasted	4.84 ± 0.60	4.37 ± 0.27	<0.0001****
1 h	9.81 ± 1.44	7.22 ± 1.34	<0.0001****
2 h	8.65 ± 1.34	6.34 ± 0.96	<0.0001****
Blood lipid level, mmol/L, mean ± SD			
CHOL	6.27 ± 1.18	6.28 ± 1.06	0.9488
TG	3.43 ± 1.51	3.17 ± 1.17	0.2105
HDL	1.7 ± 0.32	1.84 ± 0.37	0.0078**
LDL	3.27 ± 0.91	3.29 ± 0.90	0.9231
Newborn			
Gestational age, weeks, mean ± SD	38.7 ± 1.1	38.6 ± 1.0	0.6318
Birth weight, g, mean ± SD	3437.32 ± 398.96	3460.34 ± 409.38	0.6562
Macrosomia, *n* (%)	13 (11.4%)	9 (6.8%)	0.2022
Cesarean section, *n* (%)	55 (48.2%)	66 (49.6%)	0.8290
Gender			
Male	58	73	0.5290
Female	56	60	

^*a*^ Values are statistically significant at *P* < 0.05. **P < 0.01, ****P < 0.0001.

^*b*^ SD: standard deviation; GDM: gestational diabetes mellitus; PPBMI: pre-pregnancy body mass index; GWG: gestational weight gain; OGTT: oral glucose tolerance test; CHOL: cholesterol; TG: triglycerides; HDL: high-density lipoprotein; LDL: low-density lipoprotein.

### Sequencing data

The 16S rRNA gene amplicon sequencing of the meconium samples generated 20,877,882 high-quality reads after double-ended read splicing and filtering. Each sample produced at least 77,588 clean tags, with an average of 78,488 clean tags. Therefore, no samples were excluded. At a 97% similarity threshold, the clean reads were clustered via USEARCH to produce 1,328 OTUs. Ultimately, 1,187 OTUs were taxonomically annotated. The mean read length was 420 bp (ranging from 408 to 429). Rarefaction curves (Fig. S1) indicated that the number of OTUs tended to be stable when the number of reads exceeded 20,000. Thus, 25,750 sequences were randomly selected from each sample to normalize the data.

### Comparison of the neonatal gut microbiota between the GDM and control group

**Fig 1:**
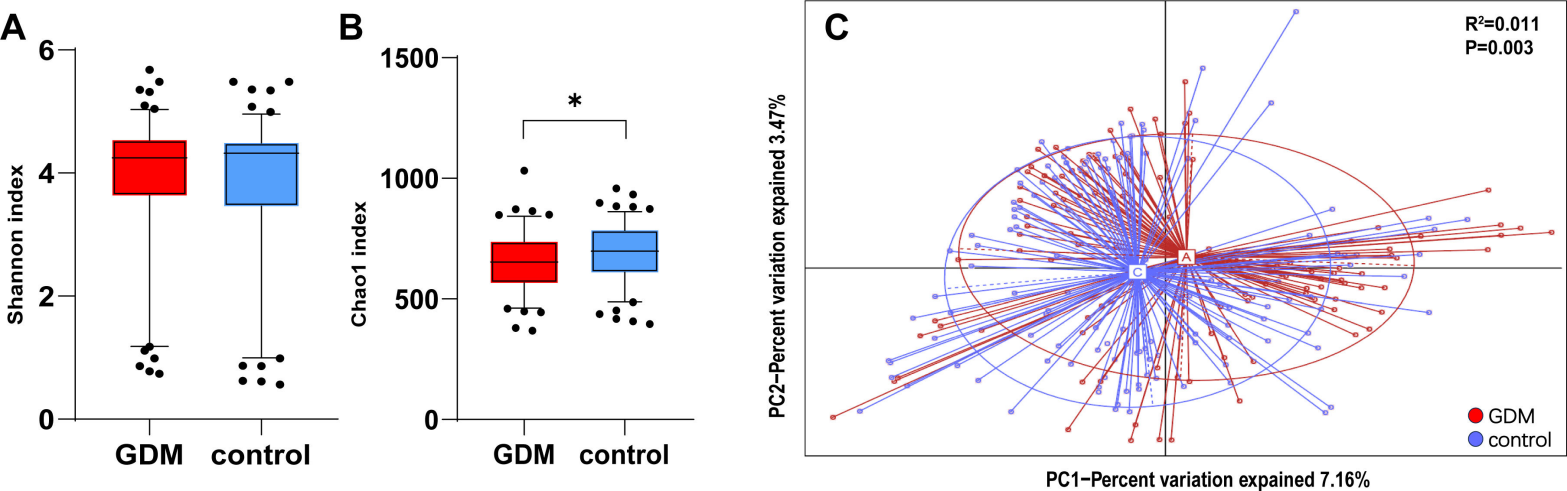
Alpha and beta diversities of the neonatal gut microbiota in the GDM and control groups. Shannon index (A) was used to assess the overall diversity, and Chao1 index (B) was used to assess the richness. Blue bars represent the control group; red bars represent the GDM group. *P < 0.05. Principal coordinate analysis of the unweighted UniFrac distances for neonatal meconium was used to assess the beta diversity (C). Ellipses represent a 95% confidence interval. Each point represents the meconium microbiota of a newborn. Blue points represent the control group; red points represent the GDM group. *R*^2^ and *P* are from PERMANOVA testing for a difference in community structure in newborns with GDM mothers compared to normoglycemic mothers.

We first investigated the composition and structure of the neonatal gut microbiota community. We did not find a difference in the number of observed OTUs between the GDM and control groups (*P* = 0.5084). The Shannon index was used to assess overall diversity, and the Chao1 index was used to thoroughly assess richness. Both indicate the alpha diversity of the meconium microbiota. The analysis revealed that the newborns from mothers with GDM presented similar diversity (Shannon, *P* = 0.7233) in their gut microbiota to that of the controls (Fig. 1A). However, the Chao1 index in the GDM group was lower than that in the control group (*P* = 0.0235) (Fig. 1B). PCoA plots based on several distances were constructed to detect differences in beta diversity. In PCoA, only with unweighted UniFrac distances (*R*^2^ = 0.011, *P* = 0.003), samples were clustered by the maternal GDM status (Fig. 1C).

**Fig 2:**
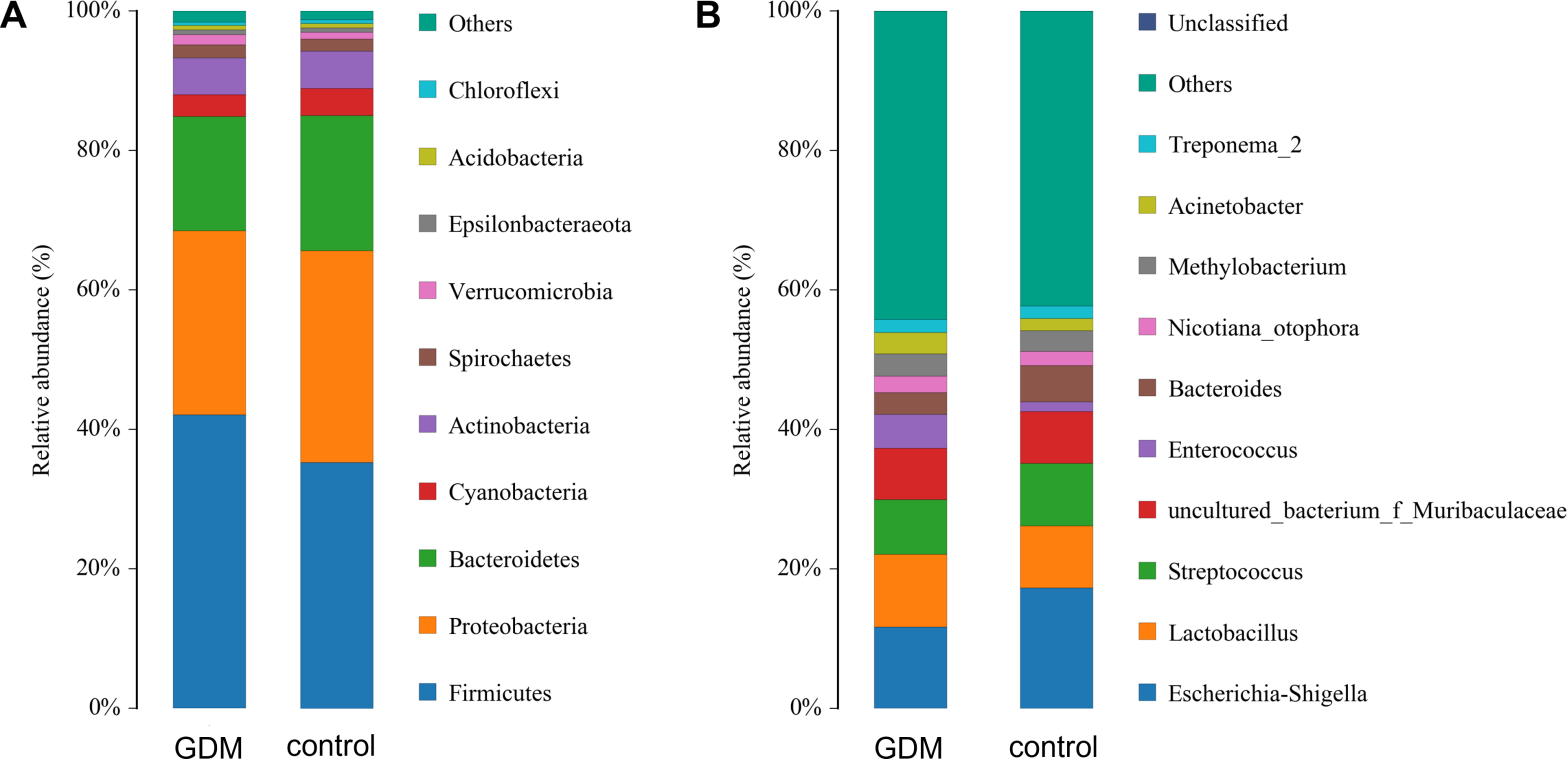
Major gut bacterial phyla and genus in the control and GDM groups. (A) Bacterial phylum average levels of each group. (B) Bacterial genus average levels of each group.

To understand the impact of GDM on individual microbes, we examined individual taxa at the phylum, class, order, family, genus, and species levels. The gut microbiota of newborns was dominated by *Firmicutes*, *Proteobacteria*, *Bacteroidetes*, and *Actinobacteria* in descending order. The composition of the phyla was similar between the two groups, whereas the *Firmicutes*/*Bacteroidetes* ratio was greater in the GDM group (Fig. 2A). The predominant genera found in both groups were *Escherichia*–*Shigella*, *Lactobacillus*, and *Streptococcus* (Fig. 2B). The genus-level compositions of the taxa were comparable between the GDM and control groups.

**Fig 3:**
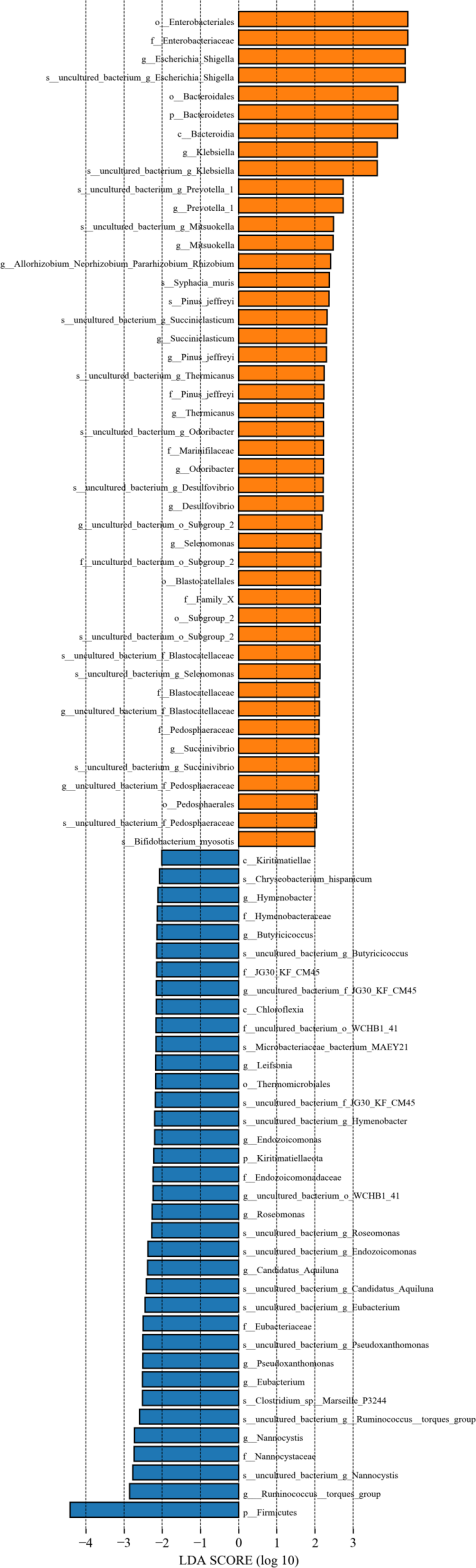
Differential taxa in meconium generated by linear discriminant analysis (LDA). Orange bars indicate bacterial taxa with significantly greater relative abundance in the control group; blue bars indicate bacterial taxa with greater relative abundance in the GDM group. LDA scores were calculated by the LDA effect size using linear discriminant analysis.

Despite the overall similarity of the microbiota structure between the GDM and control groups, the LDA indicates multiple significant representative taxa biomarkers between the groups. As shown in Fig. 3, there were 36 significant representative taxa in the GDM group and 45 in the control group. *Ruminococcus*, *Nannocystis*, *Eubacterium*, *Pseudoxanthomonas*, *Roseomonas*, *Endozoicomonas*, *Leifsonia*, B*utyricicoccus*, and *Hymenobacter* contributed to the grouping of the GDM group. In the control group, *Escherichia*–*Shigella*, *Klebsiella*, *Prevotella*, *Mitsuokella*, *Odoribacter*, *Thermicanus*, *Succiniclasticum*, *Mitsuokella*, *Selenomonas*, *Desulfovibrio*, and *Succinivibrio* were significant representative taxa.

### Influence of maternal characteristics on the neonatal gut microbiota

To determine whether perinatal characteristics influence microbial colonization in neonates, we compared the maternal BMIs, ages, GWGs, delivery modes, neonatal sexes, and neonatal birth weights between the GDM and control groups separately to explore whether these factors affected the comparison results between the GDM and control groups.

When we grouped the data according to maternal prepregnancy BMI, the alpha diversity of the gut microbiota did not show a significant difference in the GDM and control groups. However, in some subgroups, the result from PCoA revealed that the maternal GDM status clustered samples. In the maternal prepregnancy BMI ≥ 18.5 and <24 kg/m^2^ group, PCoA with binary Jaccard (*R*^2^ = 0.012, *P* = 0.002) and unweighted UniFrac distances (*R*^2^ = 0.013, *P* = 0.003) revealed that samples were clustered by the maternal GDM status (Fig. S2A). In the maternal prepregnancy BMI ≥ 28 kg/m^2^ group, PCoA with Bray Curtis (*R*^2^ = 0.070, *P* = 0.023) and weighted Unifrac distances (*R*^2^ = 0.083, *P* = 0.031) revealed that samples were clustered by the maternal GDM status (Fig. S2B). The LDA revealed 64 significant representative taxa in the GDM group and 27 in the control group according to maternal prepregnancy BMI (Fig. S3). These findings indicated that, compared with the gut microbiota of newborns from normoglycemic mothers, the gut microbiota of newborns from mothers with GDM presented more prominent taxonomic differences according to maternal prepregnancy BMI.

Maternal age (<35 and ≥35 years) did not affect the alpha diversity of the neonatal gut microbiota. In the group of maternal age < 35 years old, PCoA with binary Jaccard (*R*^2^ = 0.010, *P* = 0.0011) and unweighted UniFrac distances (*R*^2^ = 0.010, *P* = 0.026) revealed that samples were clustered by the maternal GDM status (Fig. S2C). However, GDM did not show a clustering effect on meconium microbiota in the group of maternal age > 35 years old. According to LDA, there were 41 significant representative taxa in the GDM group and 74 in the control group according to maternal age (Fig. S4).

When all the meconium samples were grouped according to insufficient maternal GWG, appropriate maternal GWG, and excessive maternal GWG, the LDA revealed that there were 20 taxa with different abundances among all the participants (Fig. S5A). However, in the GDM group, there were 27 significant representative taxa according to the maternal GWG status (Fig. S5B). In the control group, there were 50 significant representative taxa according to the maternal GWG status, and insufficient weight gain did not show significant taxa (Fig. S5C).

We also analyzed the composition of the GDM and control groups separately according to delivery mode. In the control group, the Shannon index of the gut microbiota was greater for neonates born via cesarean delivery than for those born via vaginal delivery (*P* = 0.010) (Fig. S6A). In the GDM group, there were no differences in Shannon index (*P* = 0.882) (Fig. S6B). In the control group, PCoA with Bray Curtis (*R*^2^ = 0.015, *P* = 0.045) and weighted Unifrac distances (*R*^2^ = 0.018, *P* = 0.040) revealed that samples were clustered by the delivery mode (Fig. S2D). In the GDM group, however, sample clustering shows no difference according to the delivery mode. In addition, we found that the relative abundance of *Escherichia*–*Shigella* was greater in vaginally delivered neonates both in the GDM and control groups (Fig. S7). The LDA revealed over 200 significant representative taxa according to delivery modes in the control group (Fig. S8). However, in the GDM group, there were no significant representative taxa according to delivery mode.

Neonatal sex did not affect the alpha and beta diversities of the neonatal gut microbiota. When grouped according to neonatal sex, the LDA indicated 79 significant representative taxa in the GDM group (Fig. S9A). However, in the control group, there were only seven significant representative taxa (Fig. S9B).

The newborns were divided into four groups according to their birth weight (i.e., <3,000, 3,000–3,499, 3,500–3,999, and >4,000 g). In the GDM group, there were 17 significant representative taxa among the four groups (Fig. S10A) and 32 in the control group (Fig. S10B).

The number of significant representative taxa differed greatly between the GDM and control groups according to prenatal characteristics. As shown in Table 2, maternal prepregnancy BMI and neonatal sex were associated with more significant representative taxa in the GDM group than in the control group. Maternal age, GWG, delivery mode, and neonatal birth weight contributed more significant representative taxa in the control group. In particular, the effect of the mode of delivery on biomarker taxa was significantly different between the GDM and control groups. In the GDM group, there were no significant representative taxa associated with the different delivery modes. In the control group, more than 200 taxa contributed to the LDA score according to the delivery mode.

**TABLE 2:** Number of intergroup biomarker taxa according to prenatal characteristics^*a*^

Prenatal characteristics	GDM	Control
Pre-pregnancy BMI	64	27
Age	41	74
GWG	27	50
Delivery mode	0	226
Gender	79	7
Birth weight	17	32

^*a*^ GDM: gestational diabetes mellitus; PPBMI: pre-pregnancy body mass index; GWG: gestational weight gain.

## DISCUSSION

This study revealed that newborns born to mothers with GDM had a similar composition but a different abundance of the gut microbiota compared with those born to normoglycemic mothers in our cohort. Neonates born to mothers with GDM presented a lower alpha diversity of the gut microbiota. The beta diversity results revealed that meconium samples clustered according to the maternal GDM status in some groups. In addition, maternal prepregnancy BMI, age, GWG, and neonatal delivery mode, sex, and birth weight also influenced the neonatal gut microbiota and caused significant differences in representative taxa between the GDM and control groups. The results reported in this study suggest that many other prenatal characteristics should be considered when discussing the association between maternal GDM status and neonatal gut microbiota.

The colonization of intestinal microbes after birth is a very fast process that is closely related to the living environment, feeding practices, maternal lifestyle, and maternal diet (25–28). Meconium is a unique biological sample that forms in the fetus’ gut during the 12th gestational week and reflects the earliest colonization and formation of the gut microbiota (29). Therefore, most, if not all changes may be captured with the exception of external factors. The fetal programming hypothesis proposes that exposure *in utero* may have long-term effects on health in adulthood (30). Chronic diseases in adulthood, such as hypertension, diabetes, and obesity, thus, have early developmental origins in the perinatal period. Perinatal diseases, either maternal or neonatal, such as pre-eclampsia, GDM, intrauterine growth restriction, and preterm birth, are major conditions associated with altered fetal programming and increase the risk for chronic disease in the offspring. The study of early-life colonization of the gut microbiota can help the scientific community better understand the intergenerational inheritance of diseases.

Considering the increasing prevalence of diabetes during pregnancy and its potential influence on offspring health, many scholars have studied the intergenerational genetic effects of maternal GDM on the neonatal gut microbiota. Many studies have shown that the gut microbiota of neonates born to mothers with GDM is associated with lower alpha diversity compared with that of healthy controls (15, 31). Our results revealed a lower richness (Chol 1 index) of meconium in the GDM group compared to the control group, which is in line with previous studies on meconium (16, 32, 33). Our results revealed that the relative abundance of *Firmicutes* was higher, whereas those of *Proteobacteria* and *Bacteroidetes* were lower in the meconium of newborns born to GDM mothers. However, another meconium study from South China revealed that the abundance of the phyla Proteobacteria and Actinobacteria in newborns born to GDM mothers was increased, whereas that of *Bacteroidetes* was significantly decreased^15^. Another study from China showed that *Lactobacillus* was significantly more abundant in newborns born to GDM mothers^14^. *Lactobacillus*, an important protective bacteria can inhibit the growth of harmful bacteria, such as *Escherichia* and *Salmonella*, and protect the microecological balance of the intestinal tract (34). Our study revealed no difference in *Lactobacillus* abundance between the two groups.

By comparing the participant information of these studies, we found that there were many differences in maternal perinatal factors, such as prepregnancy BMI, GWG, mode of delivery, early-life antibiotic exposure, and time of meconium acquisition. These perinatal factors have been reported to influence the neonatal gut microbiota (33, 35–38), and our study also revealed that these perinatal factors caused significant differences in representative taxa in neonates born to mothers with or without GDM. Several other perinatal factors may affect the gut microbiome, such as GWG, glycemic control in the third trimester, and insulin intervention. Previous studies did not provide comparable information about these factors, but they still cannot be ignored. This may explain why results concerning the gut microbiota vary widely across different studies. The resulting difference in the results may be more worthy of more consideration than the GDM status itself. In addition, our results show that the GDM group had lower GWG than the control group. This may be due to our hospital’s stricter management of GDM. Patients diagnosed with GDM are specifically followed up and managed by our hospital’s Perinatal Endocrinology and Metabolism Department. Pregnant women with GDM are always given individualized diet and exercise guidance by a trained dietitian (registered dietitian with an obstetrician background). The specific schedule included formulating a diet prescription according to prepregnancy BMI and physical activities. Therefore, pregnant women with GDM who receive strict management from specialists often have lower GWG compared to those who only undergo regular prenatal checkups.

Many gut microbiota, such as *Clostridium butyricum*, *Lactobacillus*, *Prevotella*, *Bifidobacterium*, *Actinomycetes*, *Ruminococcus*, and *Eubacterium*, have been identified as probiotics (39). Probiotic species can alter the population of microorganisms in the gut microbiome and regulate the function of the gut ecosystem, which has implications on digestion, immune, and central nervous system health (40, 41). We found these probiotics in both the GDM and control groups. Some taxa are also considered proinflammatory and present at relatively high levels in the meconium of infants born to mothers with T2DM (31) and in the feces of GDM patients (16, 42). Our results indicate that potential pathogenic bacteria were enriched in the neonatal gut microbiota in both groups but with different compositions. For example, *Escherichia*, *Streptococcus*, and *Bacteroides* presented high relative abundances in the control group, whereas *Klebsiella* and *Pseudoxanthomonas* were enriched in the GDM group. In conclusion, the meconium of both the GDM and control groups contained a high abundance of potentially pathogenic bacteria and probiotics but different taxa. These results suggest that effective management of GDM can reverse its effect on the neonatal gut microbiota to some extent. However, previous studies failed to explore the management effect of GDM and assess glycemic control. Therefore, further studies comparing the gut microbiota composition among non-GDM patients, GDM patients with diet modification only, GDM patients receiving insulin therapy, GDM patients with successful treatment, and GDM patients with failure of treatment are needed.

Many studies have revealed differences in gut dysbiosis in the offspring of GDM patients (14–16). However, the influence of maternal glucose metabolism disorders on the neonatal gut microbiota remains controversial. In addition to the study population differences mentioned above, the heterogeneity of the meconium samples and detection methods prevent any reliable conclusions from being drawn. To verify whether early-life colonization of the gut microbiota is a risk factor for the development of metabolic disorders in offspring, carefully designed research is needed to investigate the gut microbiota of pregnant women during pregnancy and the perinatal period and offspring during childhood, adolescence, and adulthood. Considering the increasing prevalence of diabetes during pregnancy and its potential influence on offspring health, large longitudinal cohort studies of ethnically diverse mother–child pairs with a series of maternal samples collected during the entire pregnancy, along with offspring samples collected at the early stages of life, are needed. Standardized workflows are also needed to ensure comparability and more reliable interpretations among different studies.

One strength of our study is that we set many subgroups according to many prenatal characteristics to detect their influence on the neonatal gut microbiota according to the maternal GDM status. Another strength is that we collected neonatal meconium samples within 24 h after birth, which maximally retained the intestinal microbiota state *in utero* to avoid the influence of the after-birth environment. There were also several limitations to our study. First, we analyzed only the meconium samples from newborns and did not analyze matched maternal samples. Thus, evidence connecting the fetal microbiome directly to the maternal microbiome is lacking. Second, there are still many potential influencing factors that we did not incorporate in this study, such as maternal diet, maternal physical activity level, maternal smoking status, and the management effect of GDM. Third, all of the participants were from the same hospital, and only one fecal sample per participant was analyzed. This means that some taxonomic findings may not reflect patterns in other ethnic or racial groups or other geographic regions. Future studies need large prospective cohorts of mother–child dyads from diverse racial and ethnic backgrounds to determine the direction and potential causal nature of these associations with GDM and neonatal gut microbiota. These studies should include serially collected biospecimens, standardized workflows that conserve microbial DNA and RNA, and rich data on clinical outcomes and environmental, lifestyle, and genetic risk factors for GDM.

### Conclusion

Our study demonstrates that the gut microbiota of newborns born to GDM mothers had a similar composition but different relative abundances of several taxa compared with those born to normoglycemic mothers. However, its correlation may be influenced by other perinatal characteristics. In addition to maternal diabetes status, maternal BMI, age, GWG, and neonatal delivery mode, sex, and birth weight all influenced the neonatal gut microbiota. Thus, we highlight the importance of other prenatal characteristics when discussing the influence of GDM on the initial microbial colonization of newborns. Our results provide some information for studies that aim to explore the influence of the maternal GDM status on neonatal gut microbiota and may inform future studies related to childhood disease prevention efforts based on the gut microbiota.

## Data Availability

The data supporting the results of this article have been deposited in the Sequence Read Archive (SRA; https://www.ncbi.nlm.nih.gov/sra) under the BioProject accession code PRJNA728531.

## SUPPLEMENTAL MATERIAL

Supplemental figures msphere.00914-24-s0001.pdf Figures S1 to S10.

ASM does not own the copyrights to Supplemental Material that may be linked to, or accessed through, an article. The authors have granted ASM a non-exclusive, world-wide license to publish the Supplemental Material files. Please contact the corresponding author directly for reuse.
