# Supplementary material for: Multiple perinatal characteristics affect the association between maternal diabetes status and early neonatal gut microbiota
Source: mSphere. 2025 May 16;10(6):e00914-24. doi: 10.1128/msphere.00914-24 (PMC12188708; doi:10.1128/msphere.00914-24)
Supplement: Supplemental figures — Figures S1 to S10. [file msphere.00914-24-s0001.pdf]

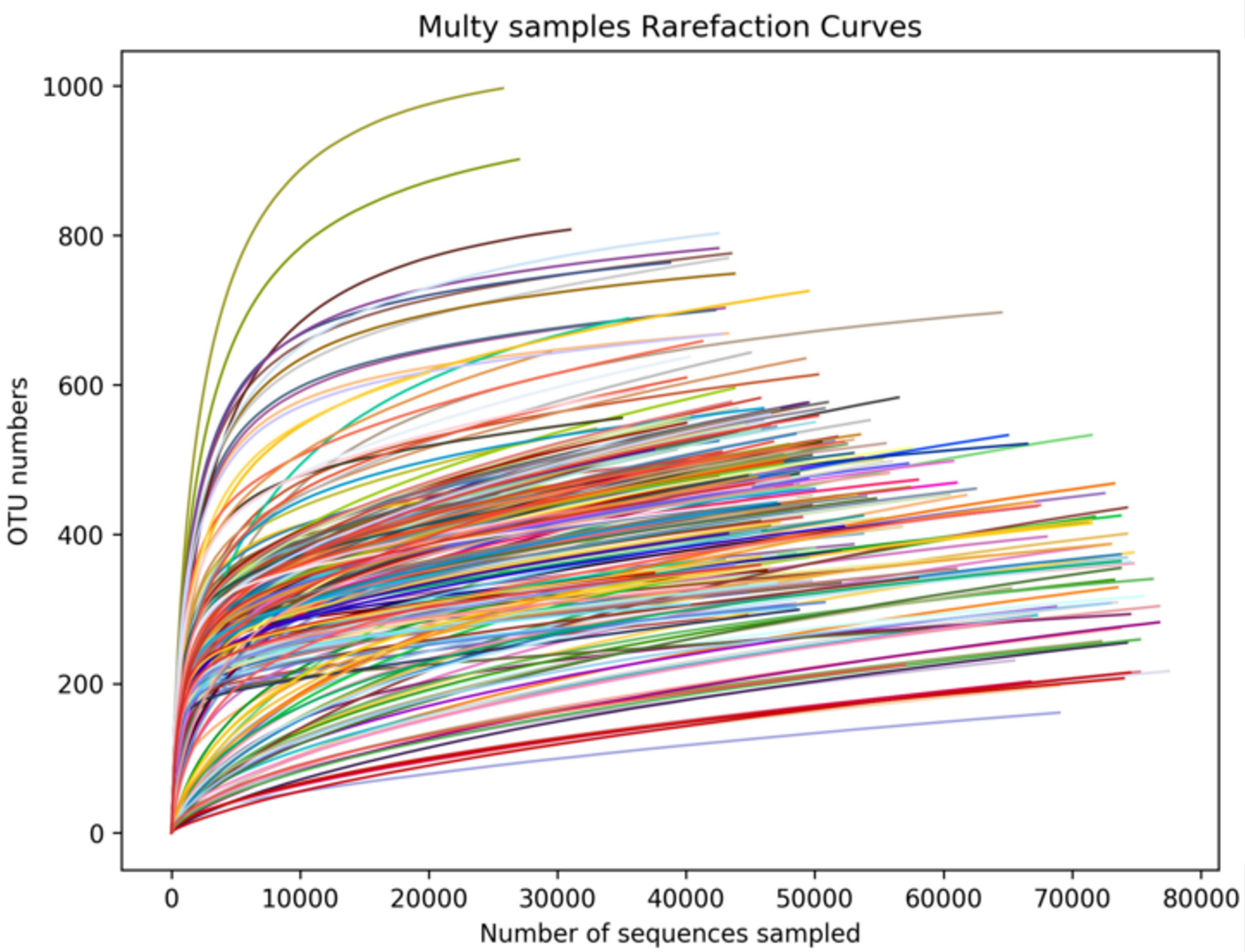

Supplementary Figure 1 Rarefaction Curves. OTU numbers tended to be stable when the number of reads exceeded 20,000.

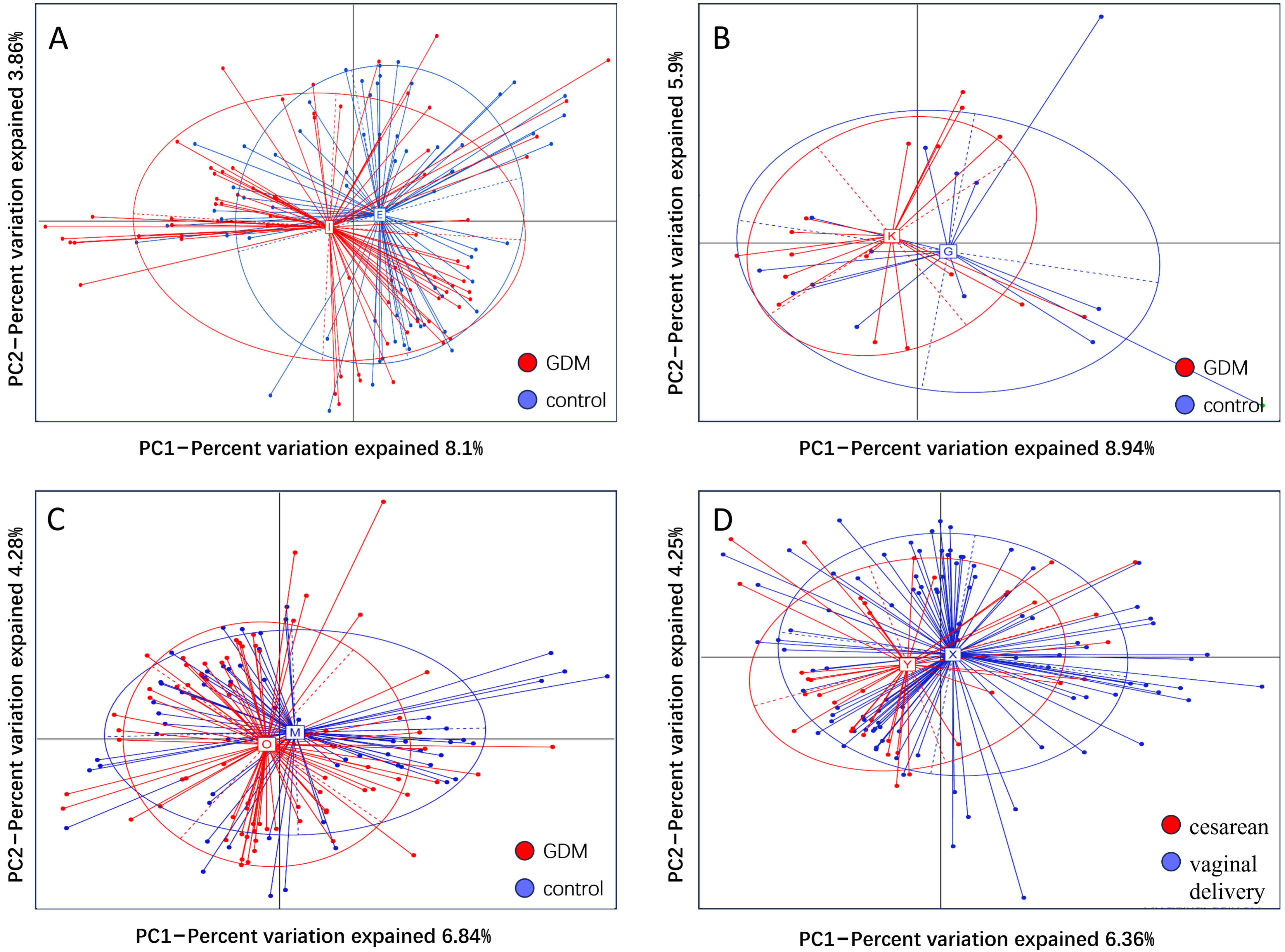

Supplementary Figure 2 Principal coordinate analysis (PCoA) for neonatal meconium was used to assess the Beta diversity. Ellipses represent a 95% CI. Each point represents the meconium microbiota of a newborn. **(A)** Maternal prepregnancy BMI⩾18.5kg/m^2 and <24kg/m^2 group. **(B)** Maternal prepregnancy BMI⩾28kg/m^2 group. **(C)** Maternal age<35 years old group. **(D)** Control group. R^2 and P are from PERMANOVA testing for a difference in community structure in newborns.

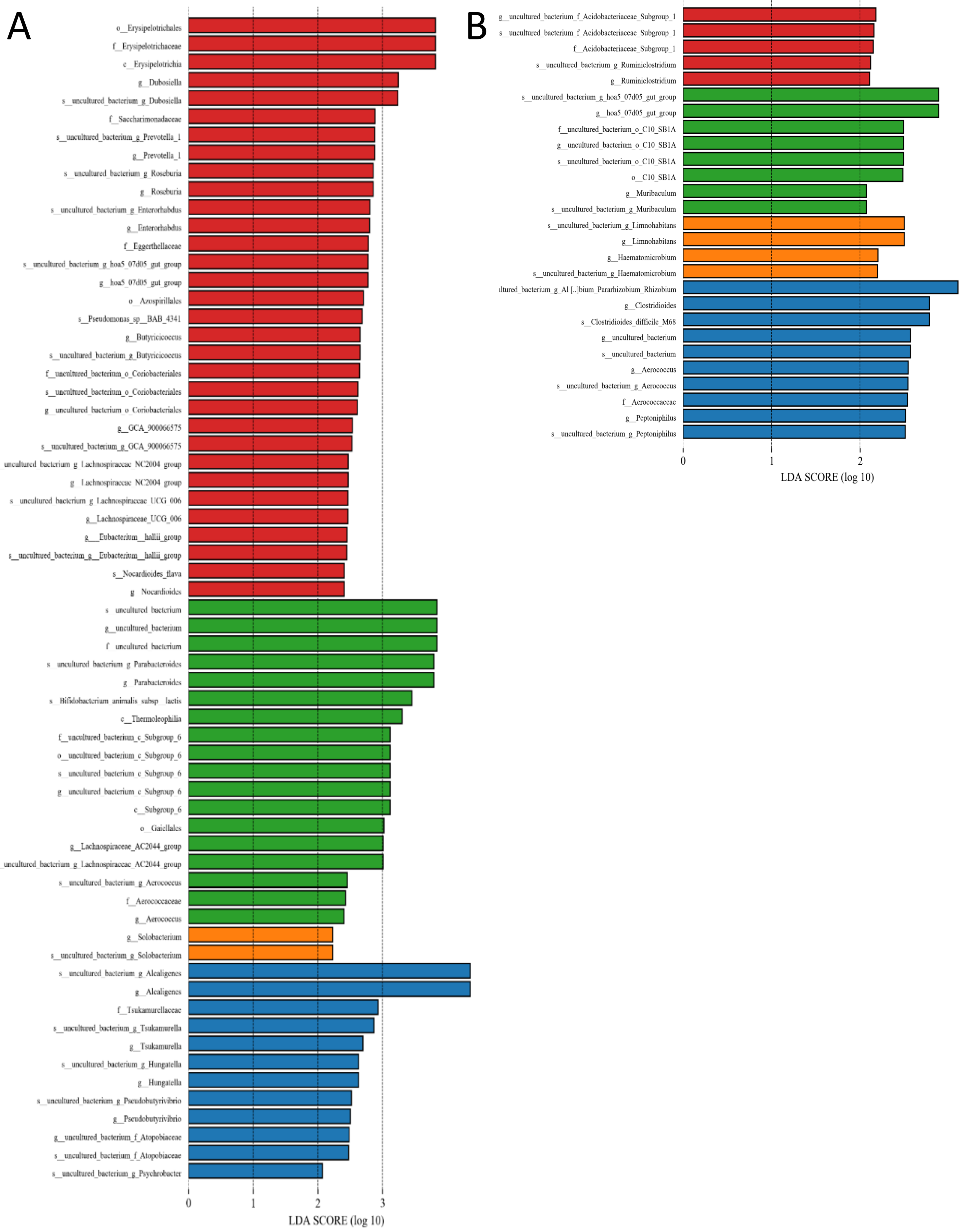

Supplementary Figure 3 Differential taxa in meconium generated by linear discriminant analysis (LDA) in the GDM group **(A)** and the control group **(B)**. Blue bars indicate maternal prepregnancy BMI <18.5 kg/m^2; yellow bars indicate BMI 18.5-23.9 kg/m^2; green bars indicate BMI 24.0-27.9 kg/m^2; red bars indicate BMI ⩾28.0 kg/m^2. LDA scores were calculated by LDA effect size using linear discriminant analysis.

A

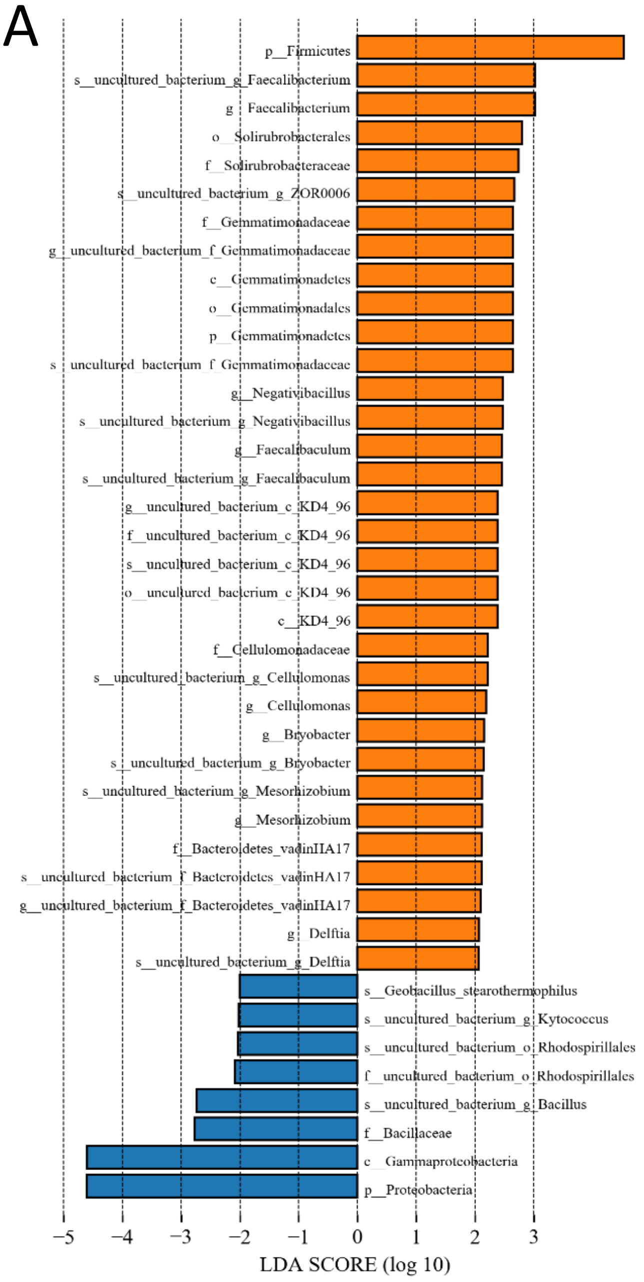

B

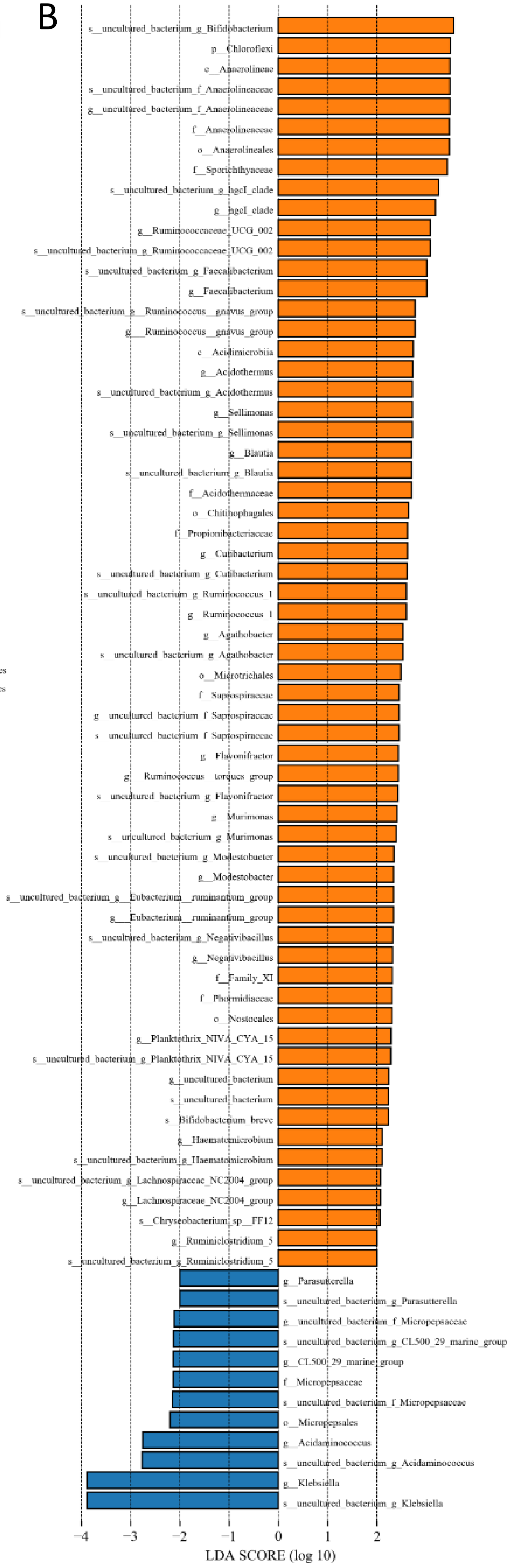

Supplementary Figure 4 Differential taxa in meconium generated by linear discriminant analysis (LDA) in the GDM group **(A)** and the control group **(B)**. Blue bars indicate maternal age≥35 years old; yellow bars indicate maternal age<35 years old. LDA scores were calculated by LDA effect size using linear discriminant analysis.

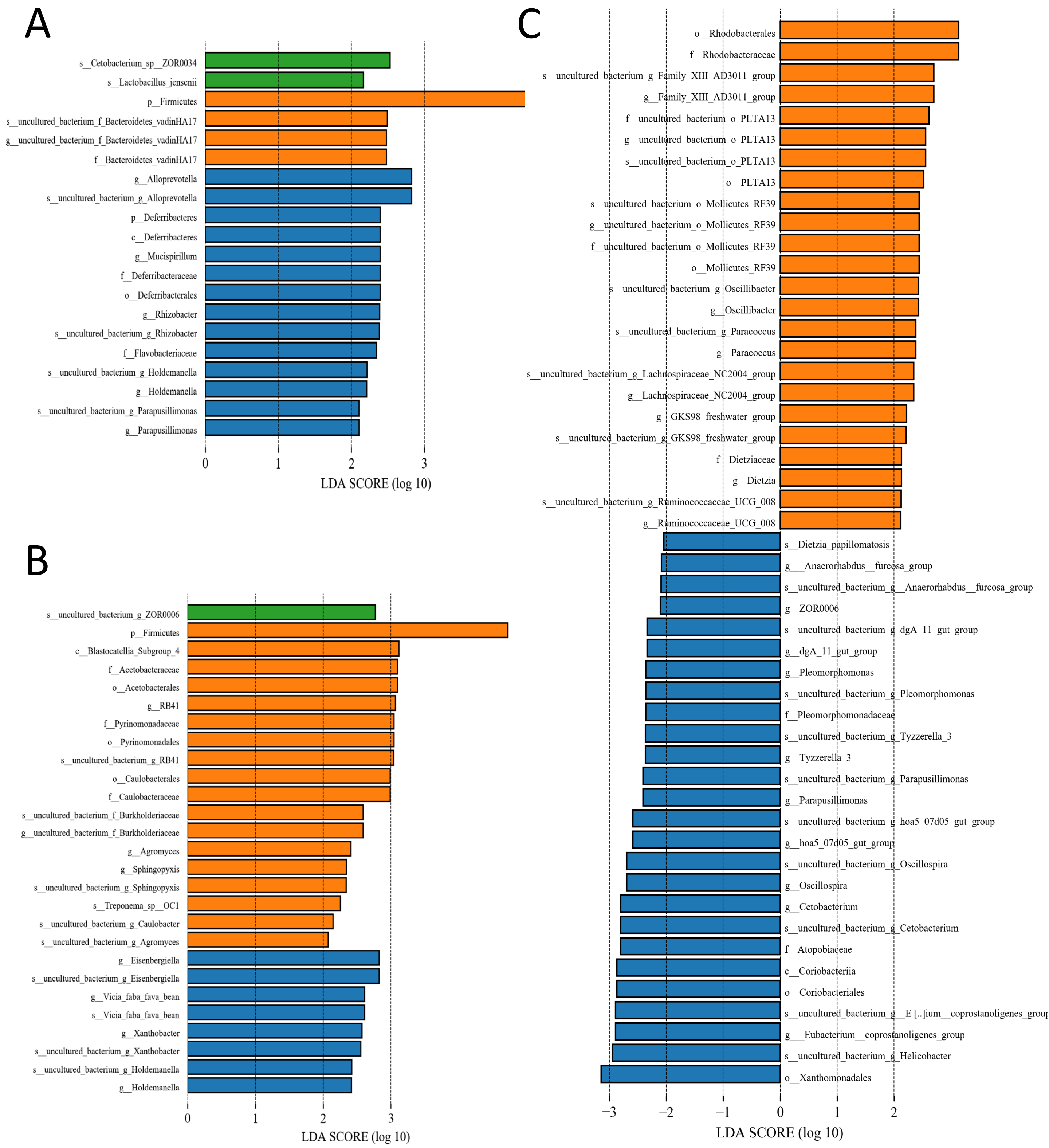

Supplementary Figure 5 Differential taxa in meconium generated by linear discriminant analysis (LDA) in all samples **(A)** and the GDM group **(B)** and the control group **(C)** according to maternal gestational weight gain. Blue bars indicate excessive weight gain; yellow bars indicate appropriate weight gain; green bars indicate insufficient weight gain. LDA scores were calculated by LDA effect size using linear discriminant analysis.

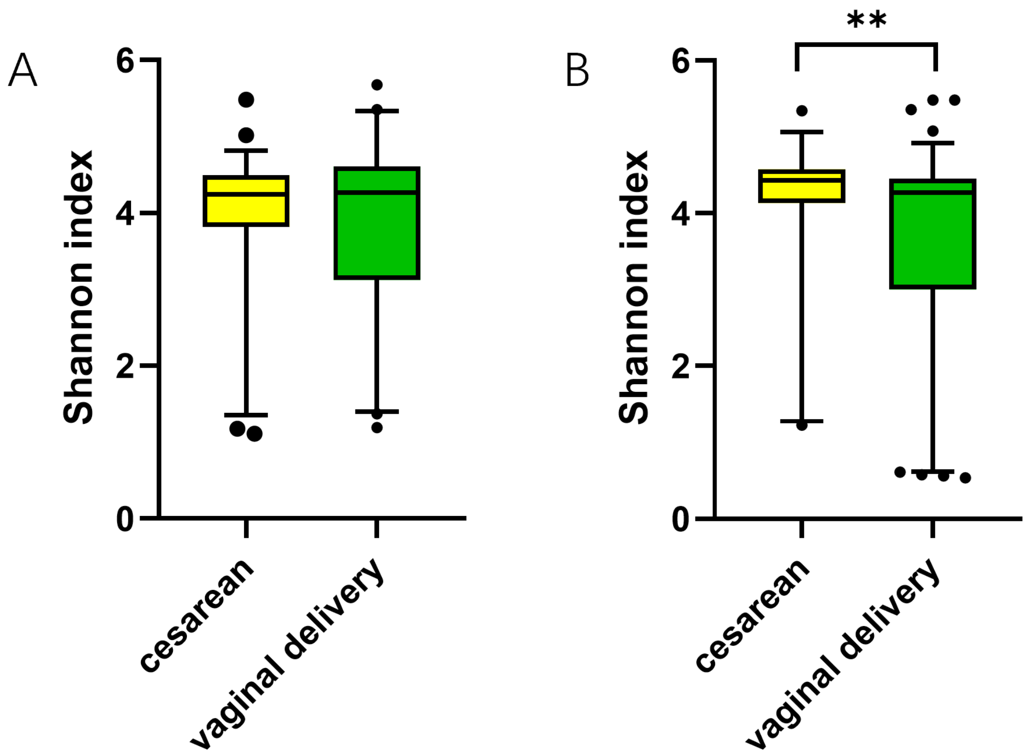

Supplementary Figure 6 The Alpha diversity (Shannon index) of the neonatal gut microbiota in the GDM group **(A)** and the control group **(B)** according to the delivery mode. Yellow bars indicate cesarean, green bars indicate vaginal delivery. (**:P=0.010)

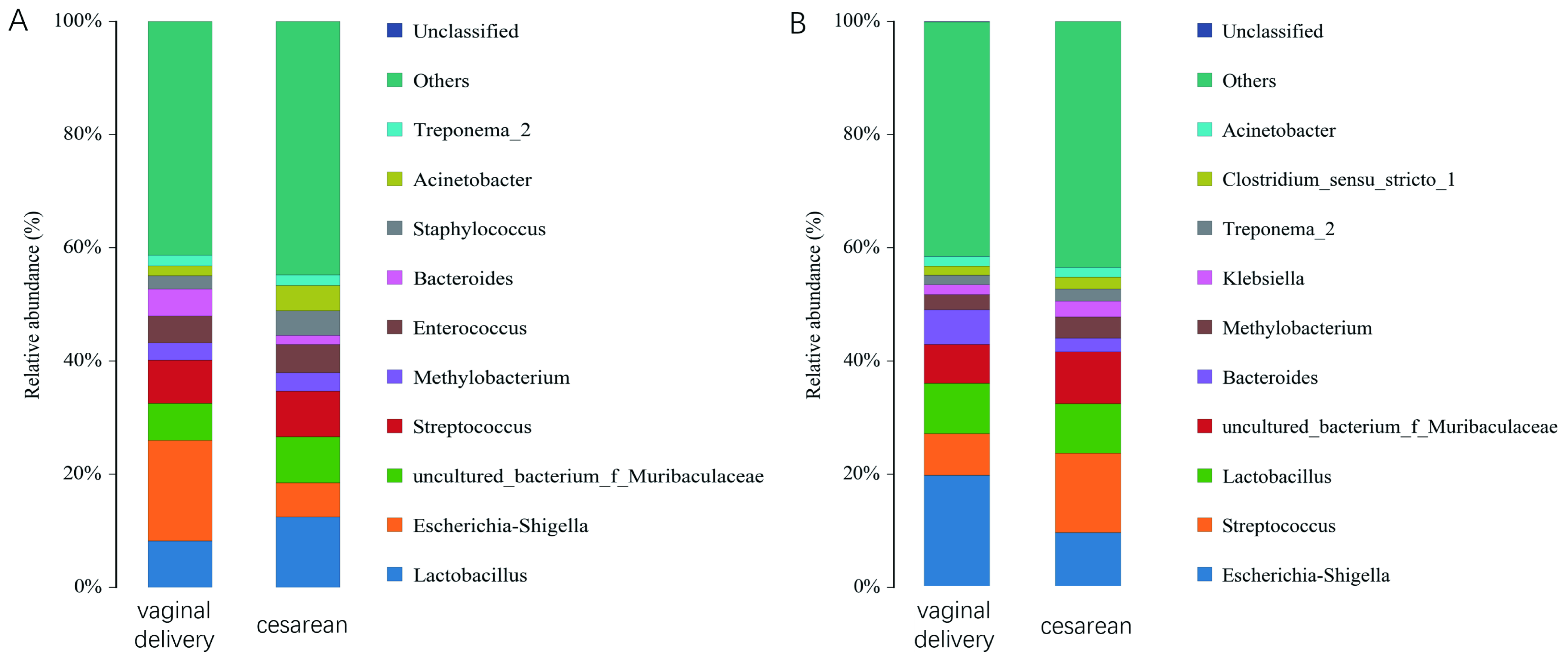

Supplementary Figure 7 The major gut bacterial genus according to the delivery mode (vaginal delivery or cesarean) . Bacterial genus average levels in the GDM group **(A)** and the control group **(B)**.

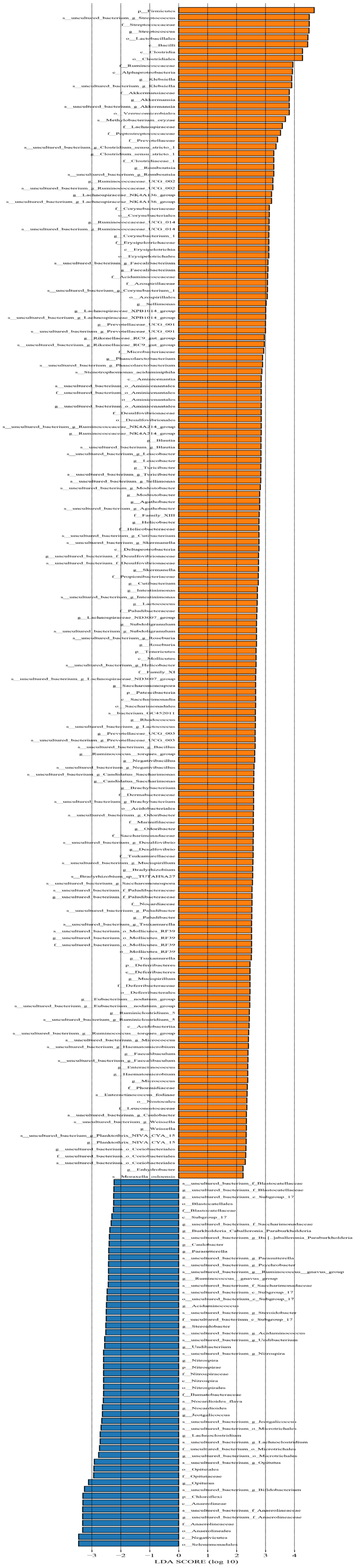

Supplementary Figure 8
Differential taxa in meconium generated by linear discriminant analysis (LDA) in the control group. Blue bars indicate cesarean; yellow bars indicate vaginal delivery. LDA scores were calculated by LDA effect size using linear discriminant analysis.

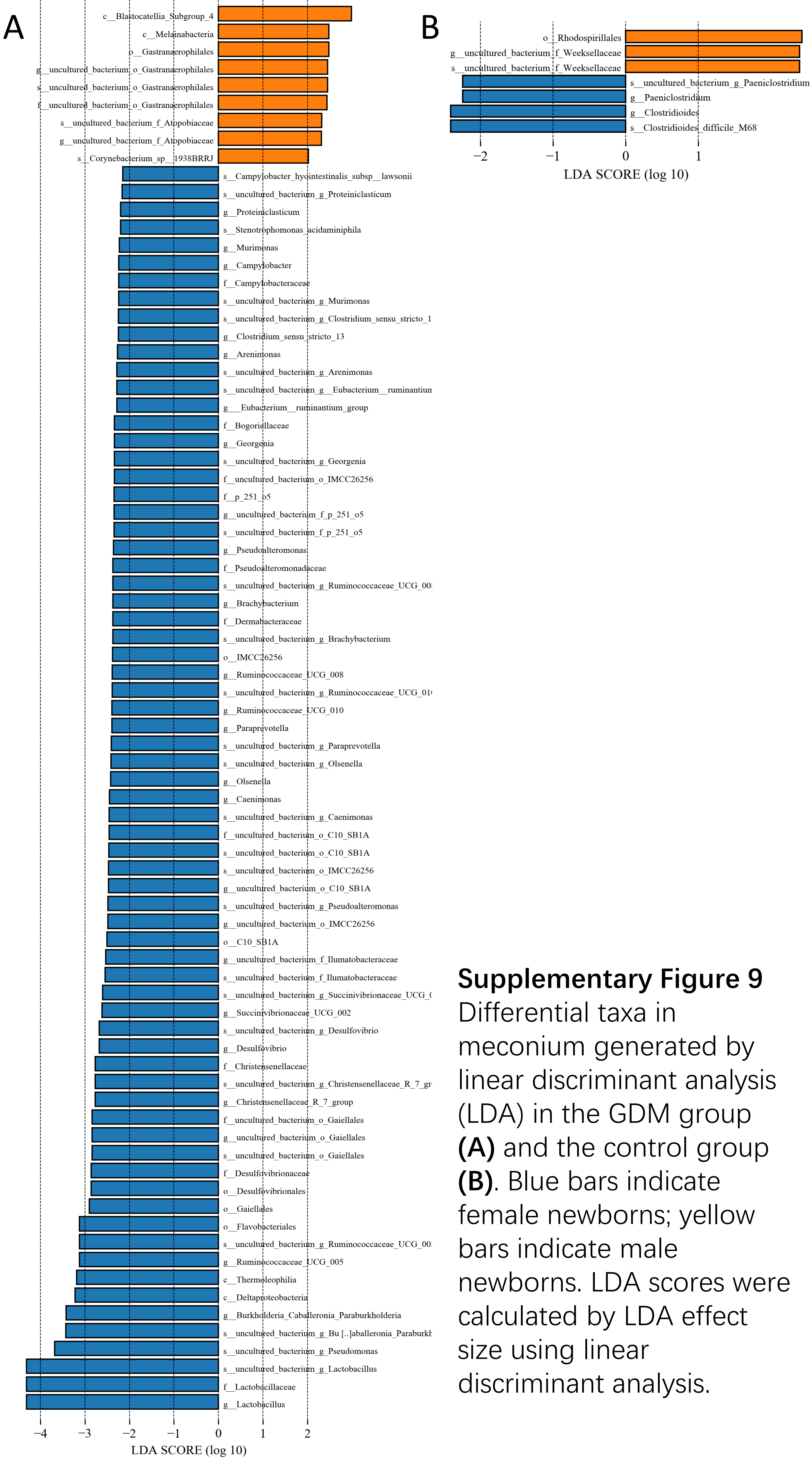

Supplementary Figure 9 Differential taxa in meconium generated by linear discriminant analysis (LDA) in the GDM group **(A)** and the control group **(B)**. Blue bars indicate female newborns; yellow bars indicate male newborns. LDA scores were calculated by LDA effect size using linear discriminant analysis.

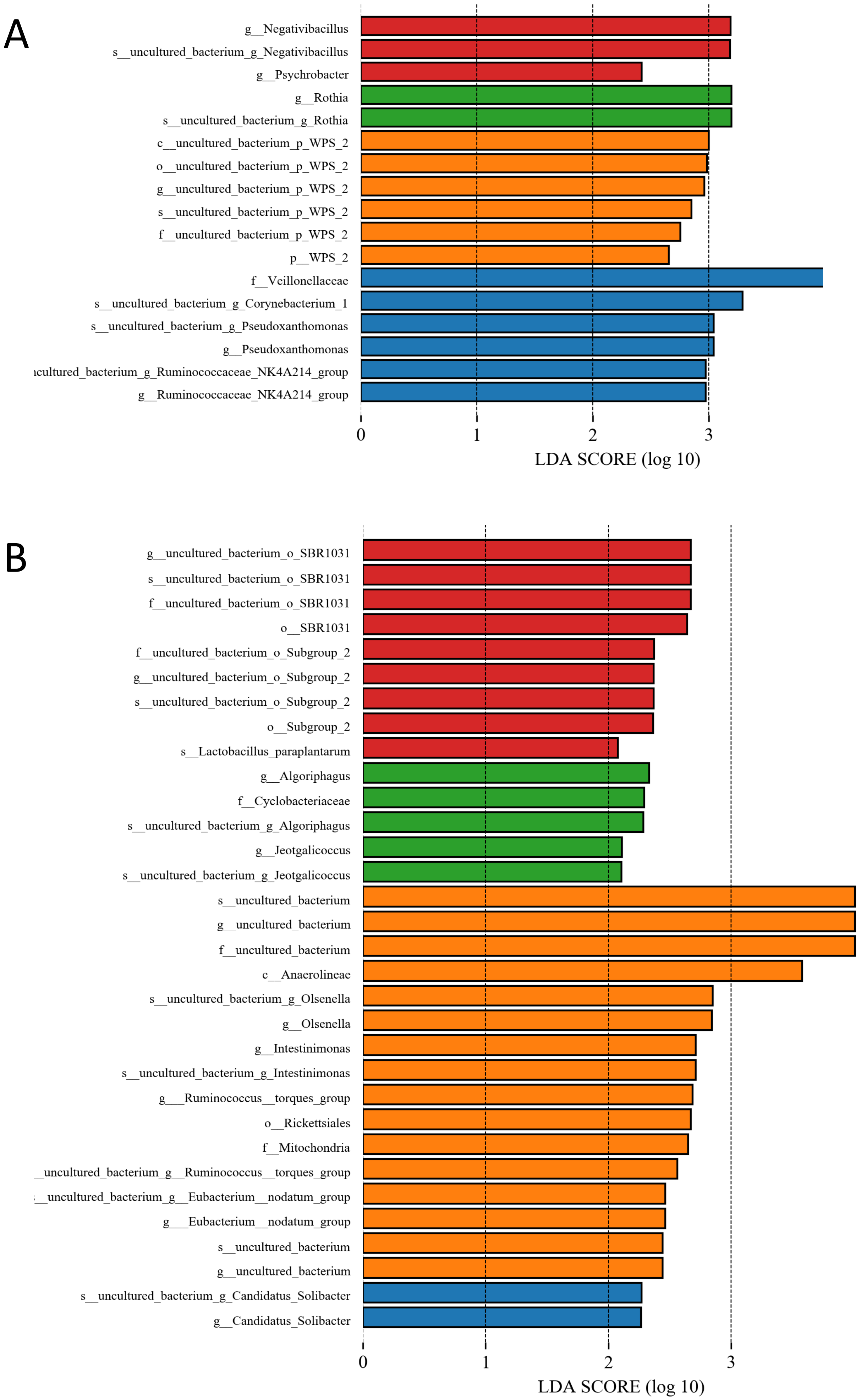

Supplementary Figure 10 Differential taxa in meconium generated by linear discriminant analysis (LDA) in the GDM group **(A)** and the control group **(B)**. Blue bars indicate neonatal birth weight⩾4000g; yellow bars indicate neonatal birth weight 3500g-3999g; green bars indicate neonatal birth weight 3000g-3499g; red bars indicate neonatal birth weight<3000g. LDA scores were calculated by LDA effect size using linear discriminant analysis.
